# Preparation of PLA/PBAT/Anthocyanins@ZIF-8 Composite Films via Casting Method and Their Antibacterial Activity and Application for Pork Preservation

**DOI:** 10.3390/molecules31172970

**Published:** 2026-08-25

**Authors:** Sheng Liu, Feifei Wang, Shuran Xing, Guifang Chang, Shijie Li, Jianwen Bu, He Zhu, Litao Wang

**Affiliations:** 1 College of Food Science and Engineering, Shandong Agriculture and Engineering University, No. 866, Nongganyuan Road, Licheng District, Jinan 250100, China; z2023066@sdaeu.edu.cn (S.L.); feiake880818@gmail.com (F.W.); xingshuran12138@gmail.com (S.X.); z2013143@sdaeu.edu.cn (G.C.); z2014011@sdaeu.edu.cn (J.B.); z2013428@sdaeu.edu.cn (H.Z.); 2 School of Pharmacy, Jining Medical University, Jining 272000, China; 13145202647@163.com

**Keywords:** anthocyanins@ZIF-8, casting method, antibacterial activity, biodegradable packaging

## Abstract

Pork is highly perishable during storage, leading to enormous economic losses and potential food safety hazards. Anthocyanins (ANTs) possess excellent antioxidant and antibacterial activities, but their poor stability in practical applications limits their industrial application. To address these issues, a novel composite preservation film was prepared by the four-sided applicator solution casting method, using polylactic acid (PLA) and polybutylene adipate-co-terephthalate (PBAT) as matrix materials and ANTs@ZIF-8 as functional filler. Zeolitic imidazolate framework-8 (ZIF-8) encapsulated ANTs to enhance its stability and achieve sustained release, while the PLA/PBAT was selected for its good biodegradability, mechanical and barrier properties. The structure and properties of the PLA/PBAT/ANTs@ZIF-8 composite films were investigated, and the results showed that ANTs@ZIF-8 nanoparticles had stable dispersibility in the matrix, which effectively improved the compatibility between nanoparticles and the film matrix. The prepared composite films exhibited excellent tensile strength (TS), elongation at break (EAB) and water vapor transmission rate (WVTR), as well as good antibacterial activity against *E. coli* and *S. aureus*. Furthermore, the practical pork preservation performance of the films was investigated, and the results demonstrated that the composite films could effectively reduce the pH value, water loss, color difference, total volatile basic nitrogen (TVB-N) content and malondialdehyde (MDA) content of fresh pork during storage, successfully extending the shelf life of pork to 12 days. This study develops a multifunctional, biosafe, and biodegradable PLA/PBAT composite film incorporated with ANTs@ZIF-8, providing a feasible strategy and scientific reference for the design and development of high-performance active biodegradable packaging materials.

## 1. Introduction

As one of the most widely consumed meat products worldwide, pork is popular among consumers owing to its abundant nutritional value, tender texture, and high protein content [1]. Nevertheless, pork is highly prone to spoilage due to its high moisture, protein, and fat contents, rendering it susceptible to microbial contamination and oxidative deterioration during storage, transportation, and marketing processes [2,3]. Pork spoilage not only results in considerable economic losses for the meat processing industry but also brings potential food safety risks to human health. The primary cause of pork spoilage is the proliferation of microorganisms, including Escherichia coli (*E. coli*), Staphylococcus aureus (*S. aureus*), and Pseudomonas, which decompose proteins, fats, and carbohydrates in pork to generate harmful substances such as total volatile basic nitrogen (TVB-N), biogenic amines, and malondialdehyde (MDA). These toxic substances not only impair the sensory quality of pork, such as its color, odor, and texture, but also may induce foodborne diseases when ingested by humans [4,5]. Thus, the development of efficient, safe, and eco-friendly pork preservation technologies is crucial for reducing economic losses, ensuring food safety, and prolonging the shelf life of pork [6]. In food packaging and preservation, conventional petroleum-based plastics including polyethylene (PE) and polypropylene (PP) remain dominant due to their low cost, excellent processability, stable mechanics, and good barrier performance. In recent years, however, growing environmental concerns and stricter plastic pollution regulations have driven the rapid development of biodegradable green packaging materials. Meanwhile, traditional preservation techniques, including physical, chemical and biological methods, suffer from high energy consumption, safety hazards and poor stability, which severely restrict their further application [7,8].

In recent years, plant-derived natural active substances have garnered widespread attention in the field of food preservation due to their safety, non-toxicity, and excellent antioxidant and antibacterial properties. Anthocyanins (ANTs), as a class of natural water-soluble flavonoid compounds widely present in plants such as red cabbage, grapes, and blueberries, possess remarkable antioxidant, antibacterial, and anti-inflammatory activities [9,10]. In the field of meat preservation, ANTs can effectively scavenge free radicals, inhibit the oxidation of lipids and proteins in meat, and restrain the growth and reproduction of spoilage microorganisms, thereby delaying the spoilage process of meat and maintaining its nutritional quality [11]. Furthermore, ANTs exert a pronounced inhibitory effect on the dominant spoilage bacteria of pork, achieving antibacterial activity by disrupting the integrity of bacterial cell membranes [12]. However, ANTs exhibit poor stability and are easily affected by external factors such as light, temperature, pH, and oxygen; such susceptibility inevitably induces structural deterioration and bioactivity attenuation during material fabrication and practical storage [13]. Critically, conventional industrial packaging technologies such as melt extrusion and film-blowing employ high-temperature processing, which triggers pronounced thermal degradation and bio-inactivation of ANTs, hindering large-scale manufacture of ANTs-based active packaging materials. Additionally, the high hydrophilicity of ANTs impedes their homogeneous dispersion within hydrophobic polymer matrices, restricting their application in food packaging and preservation films [14].

To address the aforementioned problems of ANTs, nanocarrier encapsulation technology has been extensively employed to enhance the stability and bioavailability of ANTs. Zeolitic imidazolate framework-8 (ZIF-8), a high-performance metal–organic framework (MOF) material, boasts the advantages of large specific surface area, adjustable pore structure, good biocompatibility, and pH responsiveness [15,16]. In the field of meat preservation, ZIF-8 has been regarded as an ideal carrier for natural active substances due to its unique structure and properties. Encapsulating ANTs into ZIF-8 can effectively protect ANTs from external environmental factors, reduce their degradation rate, and achieve sustained release of ANTs [17]. Furthermore, ZIF-8 itself possesses certain antibacterial activity, which can synergistically enhance the preservation effect of ANTs, providing a new approach for the application of ANTs in pork preservation [18].

With the rapid development of eco-friendly green packaging, polylactic acid (PLA)/polybutylene adipate-co-terephthalate (PBAT) binary blends featuring prominent degradability, superior physical properties and good biocompatibility were selected as the substrate material in this work [19]. Meanwhile, natural ANTs with excellent antioxidant and antibacterial bioactivities was encapsulated by ZIF-8, and multifunctional composite packaging films were fabricated by a cost-effective casting strategy for pork preservation. The microstructure, physicochemical properties, antibacterial activity and pork fresh-keeping performance of the PLA/PBAT/ANTs@ZIF-8 films were comprehensively characterized. This work aims to construct an efficient eco-friendly degradable film to retard pork spoilage and extend shelf life, providing theoretical basis and technical support for the application of biodegradable packaging materials in meat preservation.

## 2. Materials and Methods

### 2.1. Subsection

PLA, PBAT, 2-methylimidazole and methanol were purchased from Shanghai Maclin Biochemical Technology Co., Ltd. (Shanghai, China). Zinc nitrate hexahydrate (Zn(NO_3_)_2_⋅6H_2_O) was supplied by China National Pharmaceutical Group Chemical Reagent Co., Ltd. (Shanghai, China). Dichloromethane (CH_2_Cl_2_) was purchased from Yantai Far East Fine Chemical Co., Ltd. (Yantai, Shandong, China). ANTs (25% purity) were obtained from Aladdin Reagent Co., Ltd. (Shanghai, China). A total of 12 kg of fresh pork was purchased from a local agricultural market in Zibo, Shandong Province, China. Ultrapure water (resistivity > 18 MΩ·cm) was used throughout the experiments.

### 2.2. Synthesis of ZIF-8 and ANTs@ZIF-8

ZIF-8 was synthesized as follows: 1.50 g Zn(NO_3_)_2_⋅6H_2_O and 3.32 g 2-methylimidazole were dissolved in 150 mL of methanol under magnetic stirring. The mixture was reacted at 35 °C for 18 h and then centrifuged, and the precipitate was washed with methanol 2–3 times. The resulting product, ZIF-8, was vacuum-dried at 70 °C for 18–24 h, and its morphology is shown in Figure S1.

ANTs@ZIF-8 (solution A) was prepared by weighing ANTs at amounts corresponding to 1 wt%, 2 wt%, and 3 wt% of the mass of PLA/PBAT matrix, respectively, together with 0.10 g ZIF-8 (1 wt% relative to PLA/PBAT matrix). In the final composite films, ZIF-8 was kept constant at 1 wt%, while ANTs were varied at 1 wt%, 2 wt% and 3 wt% with respect to the PLA/PBAT matrix. The mixture was added to 100 mL CH_2_Cl_2_ and then magnetically stirred at room temperature for 36–48 h until no visible ANTs particles remained.

### 2.3. Preparation of PLA/PBAT/ANTs@ZIF-8 Composite Films

**Figure 1:**
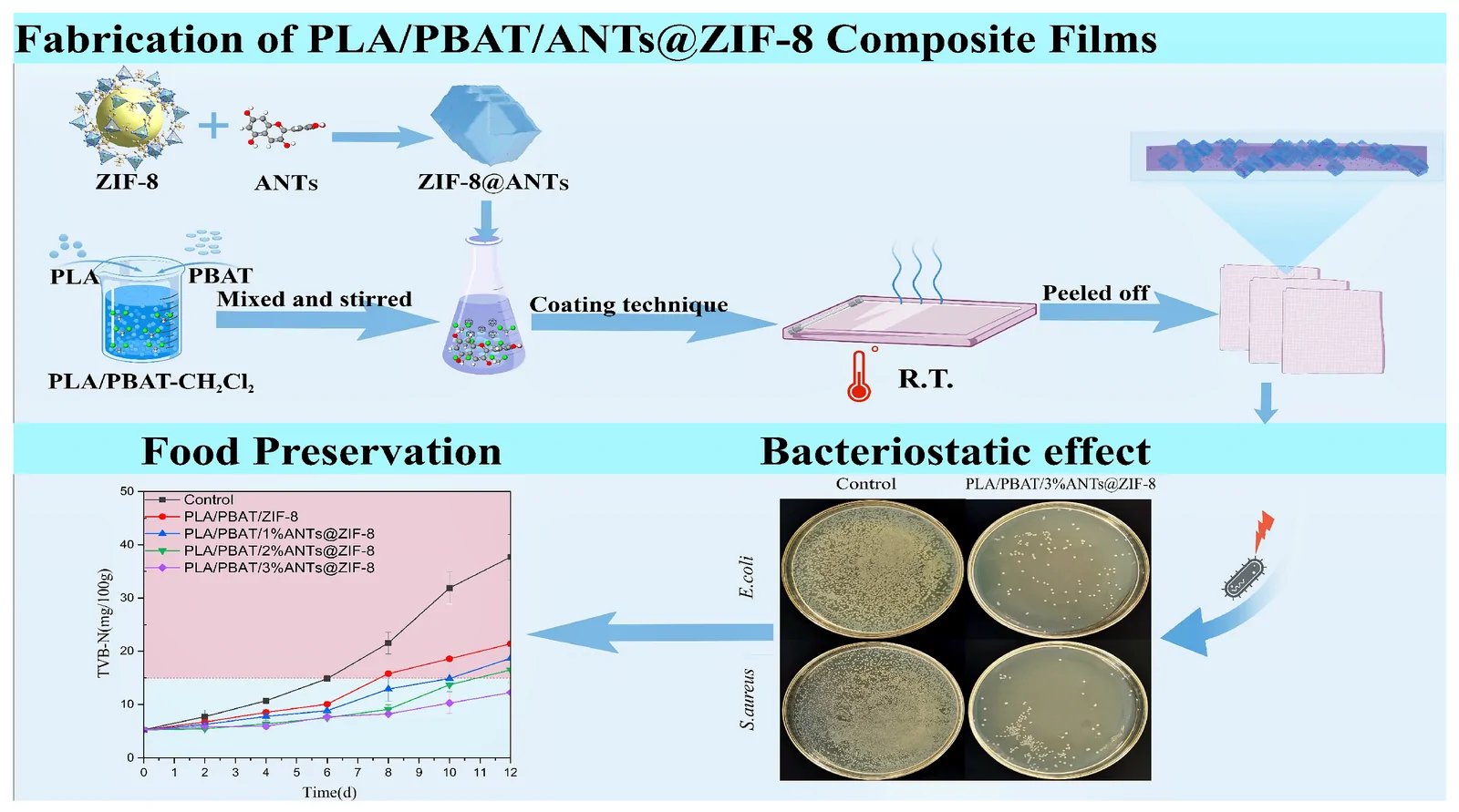
Schematic diagram of preparation of PLA/PBAT/ANTs@ZIF-8 composite films via four-sided applicator casting technique.

The preparation diagram of the PLA/PBAT/ANTs@ZIF-8 composite films is illustrated in Figure 1. The mass ratio of PLA to PBAT was determined according to the previously reported method by Xu et al. [20].

PLA/PBAT/ZIF-8 composite films were obtained as follows: First, 0.050 g ZIF-8 (1 wt% relative to PLA/PBAT) was dispersed in 100 mL CH_2_Cl_2_ and magnetically stirred at room temperature for 12–18 h. Then, 0.50 g of PLA and 4.50 g of PBAT were added to the suspension, and the mixture was magnetically stirred at room temperature for 30 min to form a homogeneous solution. The solution was casted onto a clean glass plate using a four-sided applicator (film applicator). After air-drying for 30 min, the PLA/PBAT/ZIF-8 composite film was peeled off from the glass plate.

Solution B was prepared by dissolving 1.00 g PLA and 9.00 g PBAT in 100 mL CH_2_Cl_2_. The mixture was magnetically stirred at room temperature for 30 min until a clear and transparent solution was obtained.

PLA/PBAT/ANTs@ZIF-8 composite films were prepared as follows: Solution A (ANTs@ZIF-8 suspension) was mixed with solution B and magnetically stirred for 30 min. The resulting mixture was casted onto a clean glass plate using a four-sided applicator to form a uniform thin film. The film was dried for 30 min at room temperature and then carefully peeled off from the glass plate to obtain the PLA/PBAT/ANTs@ZIF-8 composite film.

### 2.4. Material Characterization

The micromorphology of PLA/PBAT/ANTs@ZIF-8 composite films was observed by a scanning electron microscope (SEM, TESCAN MIRA LMS, Brno, Czech Republic) operated at an acceleration voltage of 10 kV. Fourier transform infrared spectroscopy (Nicolet iS5, Thermo Fisher Scientific, Waltham, MA, USA) was employed to analyze the chemical structure of the composite films within the wavenumber range of 500–4000 cm^−1^. The crystalline structures of the samples were examined by X-ray diffraction (XRD, D/MAX-2600, Rigaku, Tokyo, Japan). The XRD diffraction patterns were recorded at a scanning rate of 10°/min over a 2θ angular range of 5° to 90°. Thermal stability analysis was done using a thermogravimetric analysis (TGA, STA200, Hitachi, Tokyo, Japan). A colorimeter (SC-10, Shenzhen Sanenshi Technology, Shenzhen, Guangdong, China), pH meter (testo205, German Instrument (Shenzhen), Shenzhen, Guangdong, China), and electronic balance (JE3001, Shanghai Puchun Measuring Instrument, Shanghai, China) were used to measure the color, pH, and weight loss of samples.

### 2.5. Mechanical Properties and Water Vapor Transmission Rate (WVTR)

Tensile strength (TS) and elongation at break (EAB) of prepared composite films were tested at 25 °C using an electronic universal testing machine (WHS-10, Shandong Nake Test Equipment Co., Ltd., De Zhou, China). All experiments were performed in triplicate under consistent conditions for statistical analysis.

The WVTR of the films was determined by the cup weight-gain method according to GB/T 1037-2021 [21]. All film samples were first conditioned for 24 h under a standard environment of 23 ± 2 °C and 50 ± 5% RH. The measurement was performed at 38 ± 0.5 °C and 90 ± 2% RH. Anhydrous calcium chloride was placed inside the permeation cup to maintain a low-humidity atmosphere and establish a stable water vapor pressure gradient. After reaching a steady permeation state, the WVTR value was calculated according to Equation (1).

(1) $$WVTR=\frac{\Delta m\times 24}{A\times t}$$

where Δ*m* is the mass gain of the permeation cup (g), A is the effective water vapor transmission area of the film (m^2^), and t is the testing time (h). The unit of WVTR is g/cm^2^/48 h.

### 2.6. Antibacterial Activity

The antibacterial effects of composites films were evaluated via the turbidity method. *E. coli* and *S. aureus* were picked separately and inoculated into 20 mL of LB liquid medium. The cultures were incubated in a constant-temperature shaker at 37 °C and 180 rpm. The film samples were cut into uniform pieces and sterilized by UV irradiation (254 nm) in a clean-bench (Model SW-CJ-ZF, Boxun, Shanghai, China) equipped with a germicidal UV lamp for 30 min. Subsequently, the sterilized film pieces (2 cm × 2 cm) were immersed in 20 mL of fresh LB liquid medium, and 200 μL of the above primary seed liquid was inoculated into the medium. After thorough mixing, the mixture was incubated under the same culture conditions. Samples were taken every 2 h, and the optical density at 600 nm (OD_600_) of the bacterial suspension was determined. The bacterial growth curve was plotted according to the changes in OD_600_ values.

For the colony counting assay, single colonies of the two strains were inoculated into 20 mL LB medium containing UV-sterilized film samples, and co-cultured at 37 °C and 180 rpm for 4 h. The bacterial culture was serially diluted, and 100 μL of the dilution was spread onto glucose broth agar plates. After full absorption, the plates were inverted and incubated at 37 °C for 12 h. Finally, the colony morphology and quantity on the plates were recorded by photographing.

### 2.7. Application in Pork Preservation

#### 2.7.1. Weight Loss

After each pork sample was covered with composite films, the initial mass was recorded as *W*_1_. The mass of each sample was measured and recorded as *W*_2_ at different storage time intervals. Three parallel replicates were set for each group. The weight loss rate was calculated using the following formula in Equation (2).

(2) $$\mathrm{Weight}\;\mathrm{loss}\;(\%)=(W_{1}-W_{2})\;/\;W_{1}\;\times 100$$

#### 2.7.2. pH

Approximately 5 g pork sample was weighed, and the pH value was determined at different storage intervals using a pH meter. Before measurement, the pH electrode was calibrated with standard buffer solutions at pH 4.00 and 7.00. The electrode was inserted into the center of the pork sample. Three parallel samples were prepared for each group, and the experiment was repeated three times.

#### 2.7.3. Appearance and Color Parameters

The color parameters of pork samples were determined with slight modifications according to the method reported by Ruan et al. [22]. The lightness (L*), redness/greenness (a*), and yellowness/blueness (b*) values of the samples were measured using a colorimeter. At least five different positions were selected for each sample, and the experiment was independently repeated no fewer than three times. The total color difference (Δ*E*) was calculated by the following formula in Equation (3):

(3) $$\Delta E={\left[{\left(\Delta L*\right)}^{2}+{\left(\Delta a*\right)}^{2}+{\left(\Delta b*\right)}^{2}\right]}^{1/2}$$

#### 2.7.4. TVB-N

The TVB-N content in meat samples was determined by the automatic Kjeldahl nitrogen determination method according to the national standard GB 5009.228-2016 [23]. Briefly, a 10 g pork sample was weighed, mixed evenly with 75 mL of distilled water, and then transferred into a distillation tube and soaked for 30 min. Subsequently, 1 g magnesium oxide was added into the distillation tube containing the pretreated sample. A total of 10 mL of boric acid solution and five drops of mixed indicator were added into the receiving flask, which was quickly connected to the distillation apparatus for measurement. Three parallel samples were set for each group, and each sample was determined in triplicate to obtain the average value. The TVB-N content in the sample was calculated according to the consumed volume of standard hydrochloric acid titrant.

#### 2.7.5. MDA

The MDA detection method referred to that described by Zhang et al. with minor modifications [24]. Briefly, 5 g minced pork was mixed with 10 mL of 7.5% trichloroacetic acid and 1 mL of 0.67% thiobarbituric acid. The mixture was heated at 95 °C for 30 min and then centrifuged at 4000× *g* for 10 min. The absorbance was measured at a wavelength of 532 nm, and the results were expressed as milligrams of MDA per kg sample.

### 2.8. Data Analysis

All experimental data were expressed as mean ± standard deviation (SD). One-way analysis of variance (one-way ANOVA) followed by Tukey’s multiple-comparison test was performed for statistical evaluation. A difference of *p* < 0.05 was considered statistically significant. Origin 2021 and Excel 2021 were used for statistical analysis and data processing. All measurements were carried out in triplicate (*n* = 3).

## 3. Results and Discussion

### 3.1. Morphological and Structural Characterization of PLA/PBAT/ANTs@ZIF-8 Composite Films

**Figure 2:**
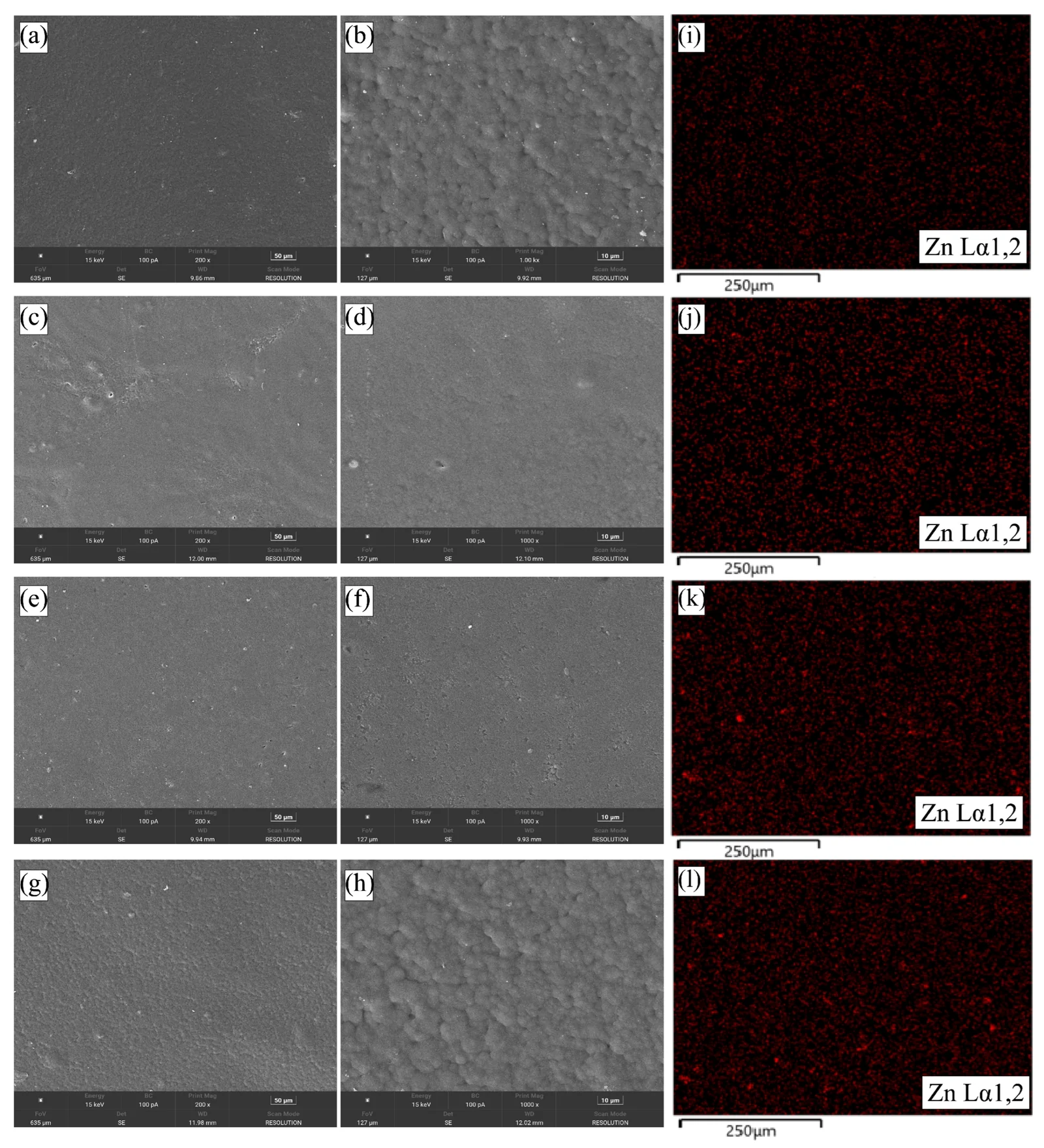
SEM surface images of different films: (**a**,**b**) PLA/PBAT/ZIF-8, (**c**,**d**) PLA/PBAT/1%ANTs@ZIF-8; (**e**,**f**) PLA/PBAT/2.0%ANTs@ZIF-8, (**g**,**h**) PLA/PBAT/3.0%ANTs@ZIF-8. (**i**–**l**): Zn elemental mapping images of different PLA/PBAT/ANTs@ZIF-8 films (ANTs: 0%, 1%, 2%, 3%).

The surface micromorphology of PLA/PBAT-based composite films was observed by SEM, as shown in Figure 2a–h. Before observation, all film samples were cryofractured and sputter-coated with a thin gold layer under vacuum. Energy-dispersive X-ray spectroscopy (EDS) was applied to acquire Zn elemental distribution maps (i–m). The PLA/PBAT/ZIF-8 film (Figure 2a,b) possesses a smooth and compact surface with distinctive spherulite-like microstructures, which originate from the intrinsic phase separation of the PLA/PBAT binary blend [25]. After the introduction of ANTs@ZIF-8 nanofillers, the resultant composite films (Figure 2c–h) exhibit well-preserved surface integrity at moderate filler loadings, with no visible cracks or large-scale agglomerates. This phenomenon demonstrates favorable interfacial compatibility between the nanofillers and polymer matrix, which is consistent with the existing literature [26]. Gradual increases in ANTs@ZIF-8 content progressively enhance surface textural roughness (Figure 2d,f,h). Notably, the PLA/PBAT/ZIF-8 film (Figure 2b) and the 3 wt% ANTs@ZIF-8 composite film (Figure 2h) exhibit highly similar coarse granular surface topographies despite their different filler compositions. The distinct morphological evolutions are primarily governed by the competitive interplay between filler–matrix interfacial adhesion and nanoparticle agglomeration behavior. Pristine ZIF-8 nanoparticles exhibit weak interfacial affinity with the PLA/PBAT matrix, thereby inducing particle aggregation and forming an inherently coarse surface structure. In contrast, moderate incorporation of ANTs@ZIF-8 (1 wt% and 2 wt%) effectively improves interfacial compatibility via ANT-mediated surface functionalization, which promotes uniform nanofiller dispersion and yields relatively smooth film surfaces. Nevertheless, excessive ANTs@ZIF-8 loading (3 wt%) induces severe nanoparticle agglomeration and deteriorates interfacial interactions. Consequently, the optimized interfacial structure collapses, and the composite surface reverts to the coarse granular morphology characteristic of unmodified PLA/PBAT/ZIF-8. Collectively, these morphological variations confirm that the solution-casting method enables uniform nanofiller dispersion under appropriate filler dosage, while excessive functional filler loading inevitably compromises interfacial homogeneity and surface quality. In the fabricated PLA/PBAT/ANTs@ZIF-8 composite films, ANTs@ZIF-8 nanoparticles exhibit excellent interfacial compatibility with the PLA/PBAT matrix, which lays a structural foundation for the stable functional performance of ANTs. The corresponding Zn elemental mapping images (Figure 2i–l) further validate the dispersion behavior of ANTs@ZIF-8 nanofillers within the polymer matrix. As a characteristic elemental marker of ZIF-8, Zn is uniformly distributed across the entire film region without obvious segregation, demonstrating the homogeneous dispersion of functional nanofillers in the PLA/PBAT blend. No obvious localized Zn-rich aggregates or large-scale clustering are observed throughout the detection area, which is highly consistent with the aforementioned SEM morphological results. This confirms the reliability and effectiveness of the solution-casting strategy in achieving uniform nanofiller distribution. Collectively, these microscopic characterizations verify that the optimized composite films possess homogeneous surface microstructure and well-dispersed nanofillers, providing favorable structural prerequisites for their subsequent functional applications.

**Figure 3:**
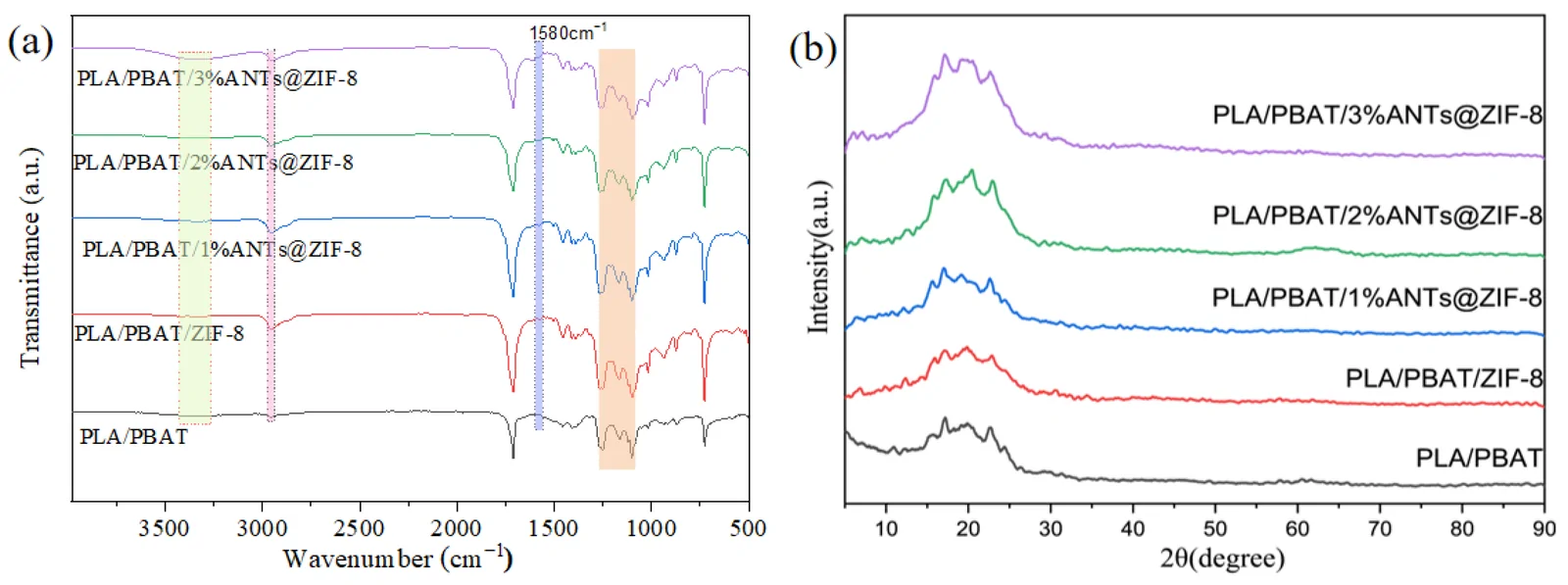
FTIR spectra (**a**) and XRD patterns (**b**) of different films.

The interactions between groups and molecules of PLA/PBAT, PLA/PBAT/ZIF-8 and PLA/PBAT/ANTs@ZIF-8 composite films were explored by FTIR spectra. As shown in Figure 3a, all samples retained the characteristic peaks of the PLA/PBAT at 3300–3600 cm^−1^ (O-H stretching vibration), ~2952 cm^−1^ (C-H stretching vibration), ~1716 cm^−1^ (C=O stretching vibration), and 1000–1250 cm^−1^ (C–O–C bending vibration), indicating the polymer structure remained intact after nanoparticle incorporation [27]. Compared with PLA/PBAT, the ANTs@ZIF-8-containing films exhibited obviously broadened and intensified hydroxyl absorption band at 3300–3600 cm^−1^ originating from -OH of ANTs, while the C=O peak at ~1740 cm^−1^ shifted slightly to lower wavenumbers (~1716 cm^−1^), confirming the formation of intermolecular hydrogen bonds between ANTs@ZIF-8 and the polymer matrix [26]. Notably, after the incorporation of a certain concentration of ANTs (2–3%), a subtle enhancement in the 1500–1650 cm^−1^ region was observed, corresponding to aromatic ring and imidazole vibrations, which confirmed the compatibility of ANTs@ZIF-8 and PLA/PBAT. These results demonstrate that ANTs@ZIF-8 was well incorporated into the PLA/PBAT matrix via hydrogen bonding.

The XRD patterns of the PLA/PBAT/ZIF-8 and PLA/PBAT/ANTs@ZIF-8 composite films are shown in Figure 3b. The broad diffraction peaks of the semi-crystalline PLA/PBAT matrix were observed at 2θ = 16–23° for all samples, demonstrating that the crystal structure of polymer matrix was maintained after incorporating ANTs@ZIF-8 [28,29]. It was also found that no typical diffraction peaks of ZIF-8 appeared in the prepared samples, which can be explained by good dispersion of ANTs@ZIF-8 nanoparticles within polymer matrix [30]. The nano-sized ZIF-8 crystallites lead to broadened diffraction signals that are overlapped by matrix peaks. With increasing ANTs@ZIF-8 loading from 1% to 3%, the intensity of the characteristic peaks of PLA/PBAT gradually increases, indicating that the ANTs@ZIF-8 nanoparticles act as nucleating agents, promoting the crystallization of the polymer matrix [31]. The absence of prominent shifts in diffraction peak positions reveals that the fillers interact with the polymer matrix predominantly through physical intermolecular forces, rather than modifying the unit-cell parameters of the polyester phase.

### 3.2. TS, EAB, WVTR and TGA of PLA/PBAT/ANTs@ZIF-8 Composite Films

**Figure 4:**
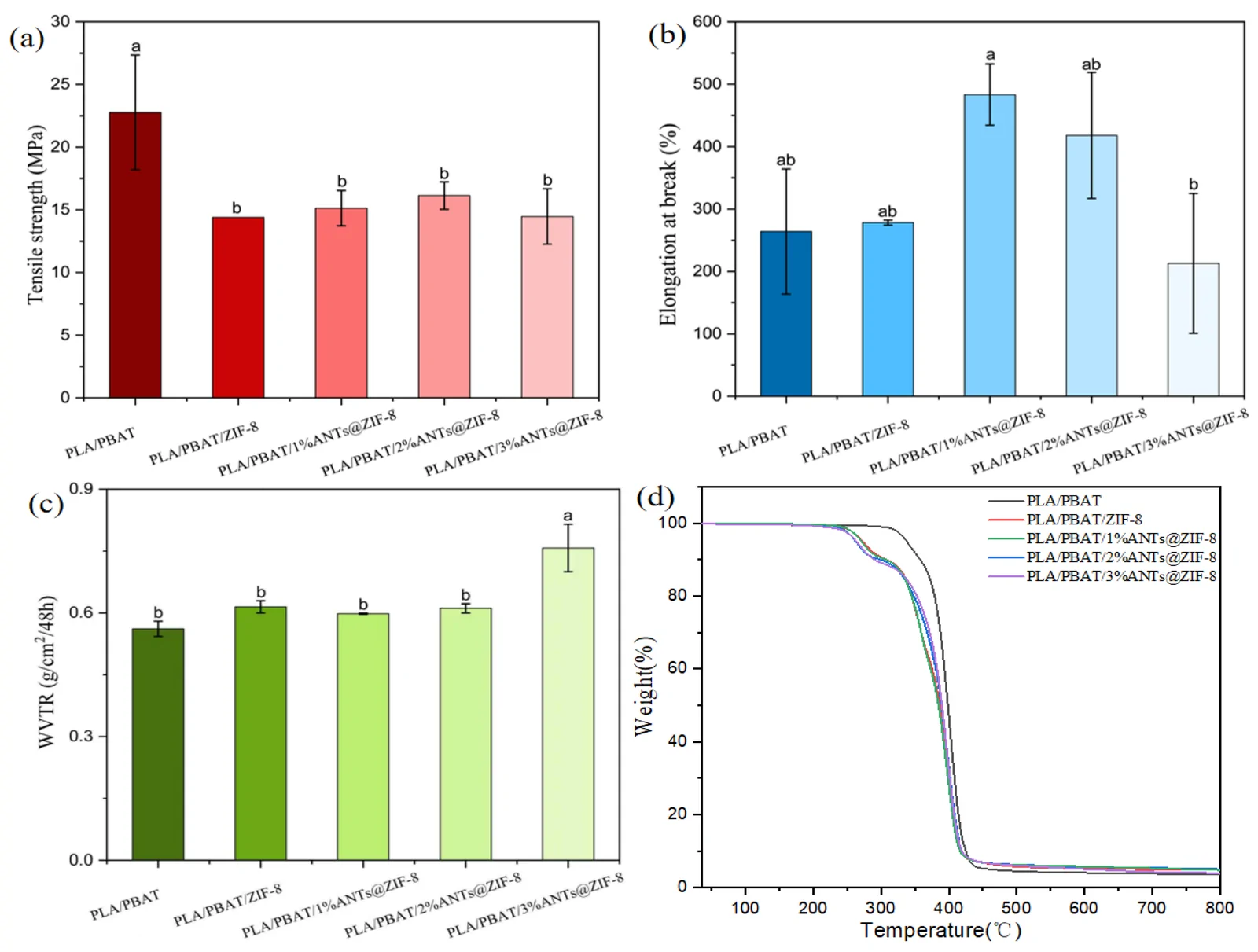
(**a**) tensile strength (TS), (**b**) elongation at break (EAB), (**c**) water vapor transmission rate (WVTR) and (**d**) thermogravimetric analysis (TGA) of different PLA/PBAT composite films. Data are expressed as mean ± SD (*n* = 3). Different lowercase letters indicate significant differences among groups (*p* < 0.05).

Excellent mechanical strength and flexible ductility are indispensable prerequisites for high-performance food packaging films, which endow packaging systems with superior structural durability against external mechanical abrasion, extrusion, and deformation during practical storage and transportation. Figure 4a,b quantitatively illustrate the TS and EAB of PLA/PBAT blend and ZIF-8/ANTs@ZIF-8 composite films, elucidating the structure–performance correlation between nanofiller incorporation and mechanical behaviors. The PLA/PBAT film exhibited the maximum TS value among all groups, which stemmed from its continuous interconnected polymer chain networks that enabled efficient interfacial stress transfer. In contrast, the incorporation of pristine ZIF-8 and ANTs@ZIF-8 nanoparticles introduced inherent interfacial voids and structural discontinuities within the polymeric matrix, disrupting the continuity of stress propagation and thereby inducing a moderate reduction in TS. All ANTs@ZIF-8-reinforced composite films maintained desirable TS above 14.0 MPa, presenting a typical volcano-type variation trend with increasing ANTs@ZIF-8 loading (1–3 wt%). At a low filler concentration of 1 wt%, ANT-mediated surface functionalization significantly strengthened filler–matrix interfacial adhesion and intermolecular interactions. The optimized interfacial microstructure effectively compensated for the structural defects induced by nanoparticle incorporation, thereby reinforcing the tensile performance of composite films [32]. However, excessive ANTs@ZIF-8 dosage (3 wt%) triggered severe nanoparticle agglomeration and phase segregation, which deteriorated interfacial compatibility, generated abundant internal stress concentration sites, and ultimately led to the pronounced degradation of TS [33].

EAB is a vital parameter for assessing the toughness and tensile deformability of packaging films, which determines their structural flexibility and deformation resistance during practical application. As illustrated in Figure 4b, all fabricated films exhibit outstanding ductility with EAB values over 200%, fully satisfying the mechanical service requirements of food packaging. Benefiting from the compact co-continuous phase structure and unimpeded polymer chain movement of PLA/PBAT matrix, pristine ZIF-8 nanoparticles can be well incorporated into the polymer network without disrupting its intrinsic deformation behavior, endowing the PLA/PBAT/ZIF-8 composite film with a ductility level comparable to that of pure PLA/PBAT. Notably, 1 wt% ANTs@ZIF-8 functionalization achieves optimal ductility among all samples. ANT-mediated interfacial modification eliminates microscale interfacial defects derived from bare ZIF-8, improves filler–matrix interfacial compatibility, and facilitates uniform polymer chain slippage under tensile stress, thereby effectively enhancing film toughness. Nevertheless, excessive ANTs@ZIF-8 loading (3 wt%) triggers severe nanoparticle agglomeration and microstructural inhomogeneity. The generated internal stress concentration sites restrict polymer chain migration and deformation, resulting in a distinct reduction in fracture elongation. Collectively, the EAB variation demonstrates that appropriate ANTs@ZIF-8 modification efficiently optimizes the toughness and flexible deformability of PLA/PBAT composite films, improving their structural stability and service durability for packaging applications.

WVTR characterizes the water vapor barrier performance of packaging films, which critically affects moisture retention and microbial growth of packaged meat during storage. Figure 4c presents the WVTR of PLA/PBAT, PLA/PBAT/ZIF-8, and ANTs@ZIF-8 composite films. The PLA/PBAT film possesses favorable intrinsic water barrier properties due to its dense polymer network. Compared with PLA/PBAT, the PLA/PBAT/ZIF-8 group shows a slightly higher WVTR (0.62 g/cm^2^/48 h), caused by interfacial voids induced by poorly compatible pristine ZIF-8 nanoparticles. By contrast, 1% and 2% ANTs@ZIF-8 modified films exhibit lower WVTR values of 0.60 and 0.59 g/cm^2^/48 h, respectively. The improved barrier performance is attributed to effective interfacial crosslinking between ANTs@ZIF-8 and the PLA/PBAT matrix, which eliminates microdefects and suppresses water vapor permeation [34]. In contrast, excessive addition of 3% ANTs@ZIF-8 significantly increases the WVTR to 0.78 g/cm^2^/48 h. The high filler loading induces severe nanoparticle aggregation, weakens filler–matrix interfacial interaction, and creates continuous water vapor diffusion pathways, thereby deteriorating the barrier performance [35]. Overall, moderate ANTs@ZIF-8 incorporation (1–2 wt%) effectively improves the water vapor barrier capability of PLA/PBAT composite films, which is beneficial for high-performance food packaging applications.

TGA (Figure 4d) was used to evaluate the thermal stability of PLA/PBAT-based composite films. No obvious weight loss was observed for all films below 200 °C, suggesting sufficient drying of the samples. All samples presented characteristic dual-stage thermal decomposition, where PLA decomposed in the first stage and PBAT degraded in the second stage [28]. The PLA/PBAT blend exhibited the optimal thermal stability. Upon the incorporation of ZIF-8, the thermal stability of the composite slightly decreased owing to the catalytic chain-scission effect of Zn^2+^ released from ZIF-8 [36,37]. For ANTs@ZIF-8-modified films, thermal stability was further reduced, which resulted from the combined action of low-temperature oxidative decomposition of anthocyanin and Zn^2+^-mediated catalysis. As the loading of ANTs@ZIF-8 increased from 1 wt% to 3 wt%, the initial decomposition temperature of films gradually shifted toward lower temperatures. Smooth and continuous TGA curves without abrupt drops demonstrated that severe large-scale agglomeration of fillers did not occur in the system.

### 3.3. Antibacterial Properties

**Figure 5:**
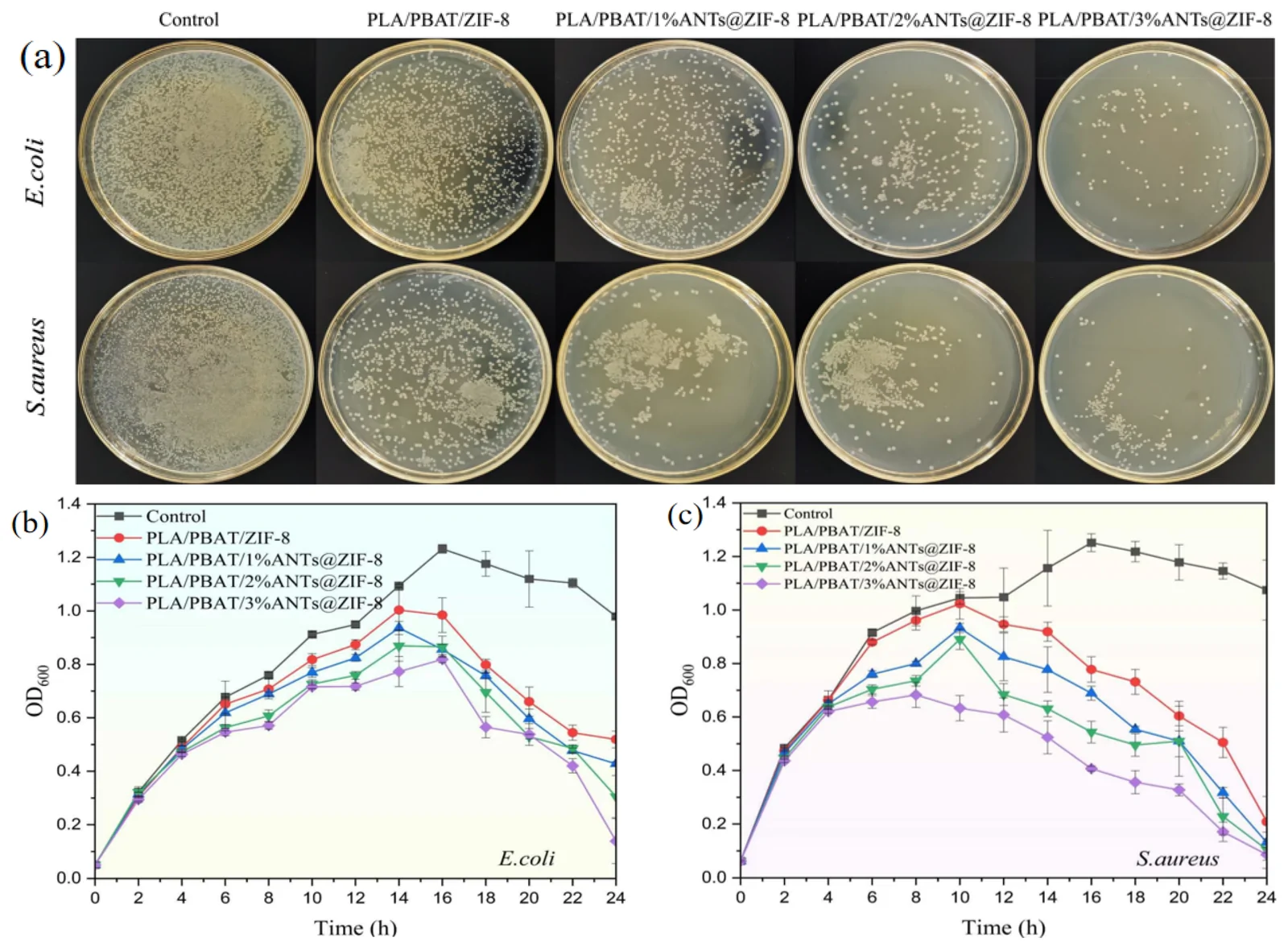
(**a**) Photographs of colony growth of *E. coli* and *S. aureus* of different PLA/PBAT-based composite films; (**b**,**c**) OD_600_-based growth curves of *E. coli* and *S. aureus* over 24 h incubation with different films. Data are expressed as mean ± SD (*n* = 3).

The antibacterial property of food preservation films can effectively inhibit the growth and reproduction of spoilage and pathogenic bacteria on the food surface, thereby delaying food spoilage and extending the shelf life [38]. Figure 5a shows the antibacterial performance of the composite films against *E. coli* and *S. aureus* by plate counting. Compared with the control group, the PLA/PBAT/ZIF-8 group showed a certain antibacterial ability, which may be attributed to the incorporation of ZIF-8. Some studies have demonstrated that the release of Zn^2+^ from ZIF-8 can contribute to antibacterial effects [39]. Meanwhile, the antibacterial activity of the prepared films against *E. coli* and *S. aureus* increased significantly with the increase in ANTs@ZIF-8 content. The enhanced antibacterial activity was attributed to the sustained release of ANTs from the ANTs@ZIF-8 nanoparticles, which damaged the bacterial cell membrane and inhibited growth [40]. This also indicates that the improved antibacterial performance of the composite films is primarily dominated by the release of ANT, rather than the Zn^2+^ ions derived from ZIF-8.

To further clarify the dynamic antibacterial behavior, 24 h bacterial growth curves were recorded. As illustrated in the bacterial growth curves (Figure 5b,c), the control group showed continuous proliferation of both *E. coli* and *S. aureus* throughout the incubation period. No obvious differences in OD_600_ were observed across all groups within the 0–4 h lag phase. During the exponential phase (4–14/16 h), the control group reached the highest growth rate, whereas moderate antibacterial suppression was achieved by the ANT-free PLA/PBAT/ZIF-8 film. Notably, increasing ANTs@ZIF-8 loading (1–3%) further depressed bacterial growth, suggesting that sustained ANTs release effectively restrained logarithmic-phase proliferation. In the subsequent stationary-decline phase (14/16–24 h), OD_600_ values declined gradually for all film-treated samples, with the 3% ANTs@ZIF-8 group delivering the strongest inhibitory effect. Originally, we attributed the superior activity toward *S. aureus* to the intrinsic susceptibility of Gram-positive bacteria to ANTs. However, the PLA/PBAT/ZIF-8 reference revealed that *S. aureus* peaked at ~10 h, in contrast to 16 h for *E. coli*. This discrepancy confirms that Zn^2+^ liberated from ZIF-8 confers preferential inhibition on *S. aureus*; lacking an outer membrane, this Gram-positive strain is more vulnerable to metal-ion damage than Gram-negative *E. coli*. Although ANTs substantially elevates the overall bactericidal capacity, the selective suppression of *S. aureus* is predominantly governed by ZIF-8-released Zn^2+^, highlighting the synergistic antibacterial action between Zn^2+^ and ANTs [41].

### 3.4. Application of Films in Assessing Pork Freshness

#### 3.4.1. Appearance

**Figure 6:**
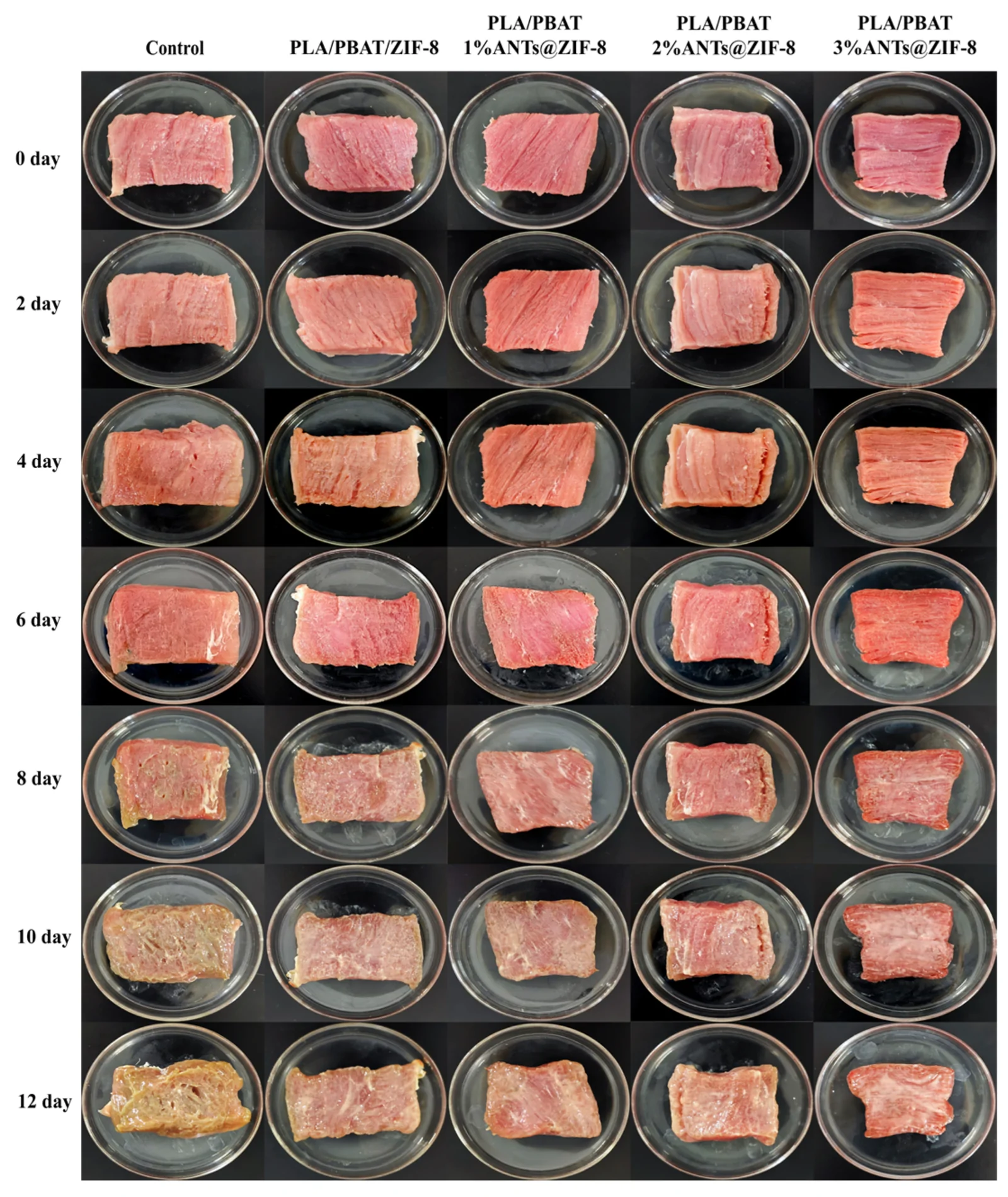
The appearance photographs of pork preservation for different storage times under different packaging materials.

The preservation effect of the prepared composite films on fresh pork was evaluated (Figure 6). The color changes of pork samples wrapped with different PLA/PBAT/ANTs@ZIF-8 composite films were observed during 12 d storage. At the initial stage (0–4 d), all pork samples exhibited bright pink color with no obvious difference. The control group showed slight darkening, while the film-wrapped groups maintained favorable redness. From 6 to 8 d, obvious color differences were observed. The control group turned grayish and brown due to myoglobin oxidation and microbial spoilage. With the increase in ANTs@ZIF-8 content in PLA/PBAT/ANTs@ZIF-8 composite films, the composite films effectively retarded browning, with the 3% ANTs@ZIF-8 group retaining the brightest red color. In the final storage period (10–12 d), the control group emerged severe yellow-brown discoloration and spoilage. By contrast, the 3% ANTs@ZIF-8 film group exhibited the best color preservation performance, which was attributed to the antioxidant activity of ANTs and the strong antibacterial effect of the composite film, slowing down myoglobin oxidation and microbial-induced spoilage [42].

#### 3.4.2. pH

**Figure 7:**
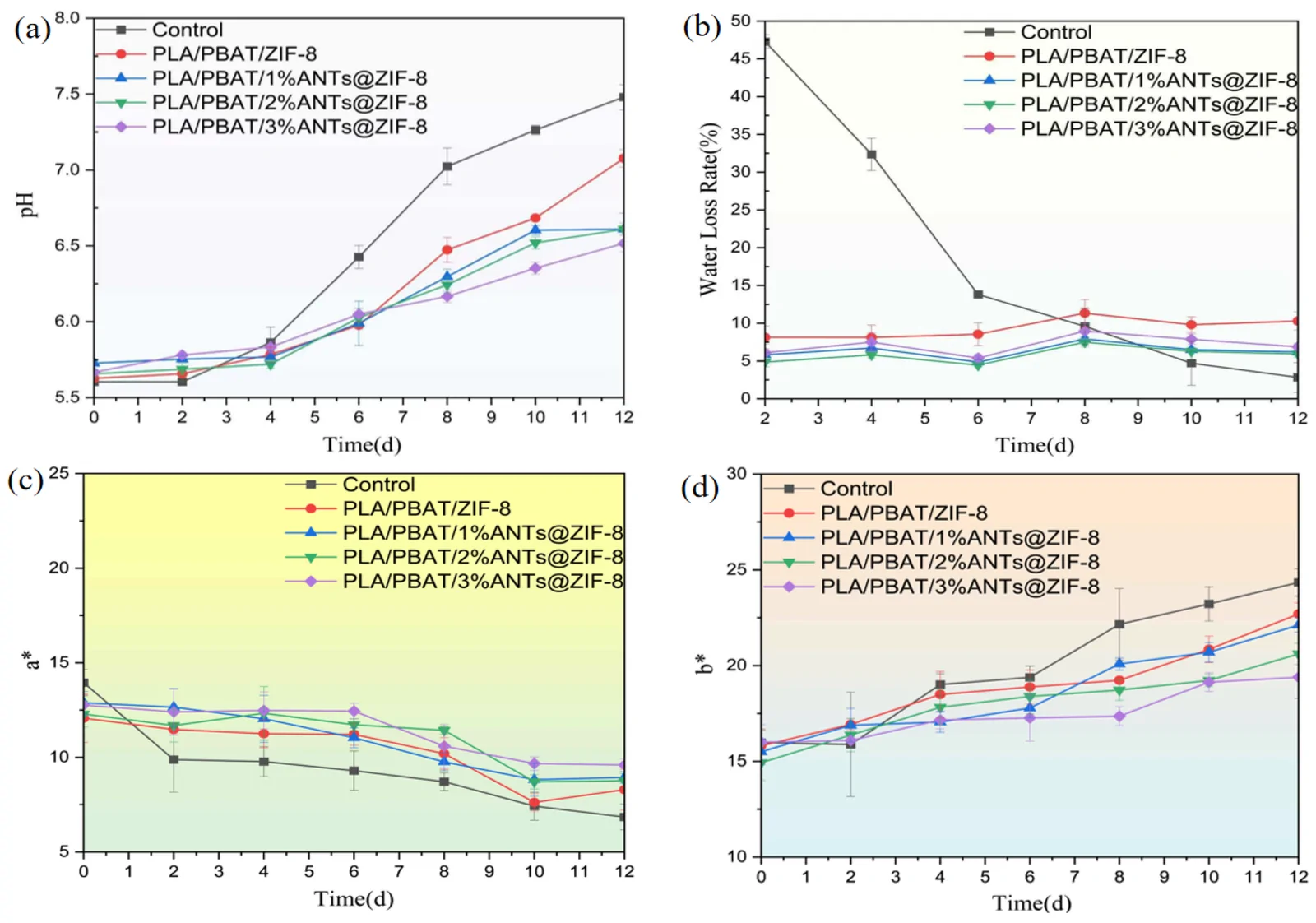

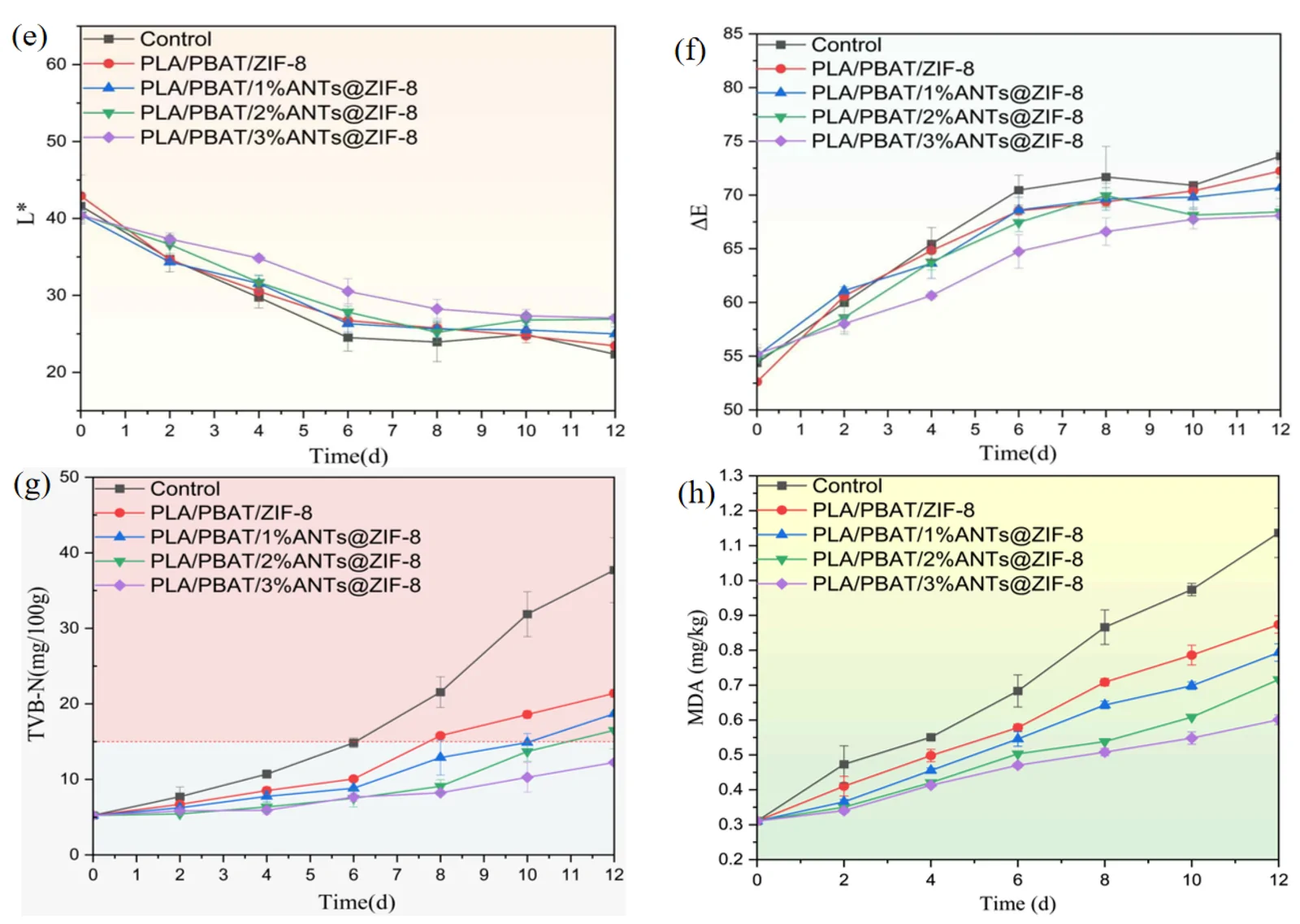
Changes in pH (**a**), water loss rate (**b**), a* (**c**), b* (**d**), L* (**e**), Δ*E* (**f**), TVB-N (**g**) and MDA (**h**) value of pork samples during storage at 4 ± 1 °C. Data are expressed as mean ± SD (*n* = 3).

The pH changes of fresh pork samples wrapped with PLA/PBAT/ANTs@ZIF-8 composite films during storage were shown in Figure 7a. Overall, the pH value of all pork samples gradually increased during 12 d storage, which is mainly attributed to the production of alkaline substances such as ammonia and biogenic amines from microbial metabolism and protein degradation in spoiled meat [43]. It was also observed that the composite films effectively slowed pH rise in pork with the increase in ANTs@ZIF-8. In the early storage stage (0–4 d), the pH values of all groups remained stable at around 5.6–5.8, and there were no obvious differences observed among groups. From day 4, the pH of the control group increased rapidly and reached the highest value of approximately 7.5 on day 12, indicating accelerated protein degradation and rampant microbial spoilage. The PLA/PBAT/ZIF-8 group showed a slower pH increase than the control group due to the mild antibacterial effect of films. Microbial growth and metabolism induce the production of large amounts of spoilage metabolites during storage [44]. The introduction of ANTs@ZIF-8 markedly inhibited the rapid rise in pork pH, further confirming that ANTs plays a crucial role in inhibiting meat spoilage. Among all treatments, the PLA/PBAT/3%ANTs@ZIF-8 group exhibited the slowest pH elevation, maintaining the lowest pH value throughout the whole storage period. This phenomenon can be explained by the strong antibacterial activities of ANTs, which inhibited microbial growth and protein degradation, thus delaying the accumulation of alkaline metabolites in pork [45].

#### 3.4.3. Weight Loss

Water loss rate is a critical indicator reflecting the water-holding capacity and freshness of pork during storage [46]. As shown in Figure 7b, the control group exhibited an extremely high early water loss rate, which decreased rapidly from day 2 to day 8 and remained low in the later stage, mainly due to rapid free-water evaporation in the early storage period. In contrast, pork samples wrapped with PLA/PBAT-based composite films displayed significantly lower water loss rates throughout the whole storage period. The PLA/PBAT/ZIF-8 group showed a moderate water loss rate, while the incorporation of ANTs@ZIF-8 further improved the water-retention property of the films. Among all composite film groups, the PLA/PBAT/2%ANTs@ZIF-8 group achieved the lowest water loss rate, indicating the best water barrier property. This is because the dense structure of composite films effectively blocks water vapor permeation, and ANTs@ZIF-8 nanoparticles improve the compactness of the film matrix, thus reducing moisture evaporation from pork and maintaining its juiciness. However, it was also found that the water loss rate of the PLA/PBAT/3%ANTs@ZIF-8 group was higher than that of the other groups, and this result deviates from WVTR data of standalone films (Figure 4c). Measured under standardized vapor–pressure gradients, WVTR reflects the intrinsic steady-state permeability of bare membranes. Elevated WVTR at 3% ANTs@ZIF-8 stems from filler-aggregation-induced micro-voids, whereas MOF-constructed tortuous pathways partially counteract defect-driven permeation at 3% loading, yielding slightly reduced yet still higher WVTR than neat PLA/PBAT. By contrast, storage water-loss rate represents coupled water migration in the non-equilibrium film-meat system. Despite its relatively high intrinsic permeability, the 3% composite sequesters pork-released water via ZIF-8 frameworks. This water-retention effect suppresses macroscopic water escape and accounts for its unamplified water loss during preservation.

#### 3.4.4. Color Parameters

Color parameters (a*, L*, b* and Δ*E*) were used to characterize the color stability of pork packaged with PLA/PBAT-based composite films. As shown in Figure 7c, the a* value (redness) continuously declined with storage time because of oxymyoglobin oxidation to metmyoglobin across all treatments [47]. The control group lost redness most rapidly, while ANTs@ZIF-8-modified films effectively maintained pork redness. The b* value (yellowness, Figure 7d) increased in all samples owing to lipid oxidation, and the 3% ANTs@ZIF-8 group exhibited the lowest yellowness increment. The L* value (lightness, Figure 7e) gradually decreased in all groups during storage due to myoglobin oxidation, and the control group showed the sharpest decline. The PLA/PBAT/ANTs@ZIF-8 composite films retained higher lightness, especially the 3% ANTs@ZIF-8 group. Total color difference Δ*E* (Figure 7f) increased steadily over storage, with the control group presenting the highest color deterioration. The composite films obviously reduced pork color variation, and the PLA/PBAT/3%ANTs@ZIF-8 group achieved the minimum Δ*E*. These findings verified that ANTs successfully inhibited myoglobin oxidation and lipid peroxidation, thus stabilizing pork color and improving its visual quality during preservation.

#### 3.4.5. TVB-N

TVB-N is a critical indicator to evaluate meat spoilage, which originates from microbial-mediated protein degradation [48]. As shown in Figure 7g, the TVB-N values of all pork samples increased continuously during storage. The control group exhibited the fastest TVB-N accumulation, exceeding the acceptable freshness threshold (15 mg/100 g) on day 6 and reaching the highest level on day 12, indicating noticeable microbial spoilage. In contrast, PLA/PBAT-based composite films significantly retarded the rise in TVB-N. The TVB-N-inhibiting effect was enhanced with increasing ANTs@ZIF-8 content, and the PLA/PBAT/3%ANTs@ZIF-8 group kept the lowest TVB-N value below 15 mg/100 g over the whole 12-day storage period. Various biodegradable active packaging films have been developed for chilled pork preservation in recent studies. Cinnamaldehyde-loaded chitosan film extended the pork shelf life for only six days at 4 °C [49]. ZIF-67-modified loquat seed starch/pectin composite film achieved an eight-day preservation period under the same storage condition [50]. By comparison, curcumin-based gelatin/serum-plasma biofilm could maintain pork freshness for 12 days [51]. In this work, the optimized PLA/PBAT/3%ANTs@ZIF-8 composite film also realized a 12-day shelf-life extension of chilled pork at 4 °C, exhibiting superior and competitive preservation efficiency over most reported biopolymer-based packaging materials. Furthermore, the PLA/PBAT matrix provides better mechanical properties, water resistance and processing adaptability than traditional chitosan, gelatin and starch-based films, demonstrating greater potential for practical food packaging application. These excellent preservation performances are attributed to the synergistic antibacterial and antioxidant effects between ANTs and ZIF-8 nanoparticles. The synergistic system effectively inhibits microbial reproduction and reduces protein oxidation and decomposition in pork, significantly retarding the spoilage process and achieving a prominent shelf-life extension effect.

#### 3.4.6. MDA

MDA is a typical secondary product of lipid oxidation, which reflects the degree of lipid peroxidation in pork during storage [52]. As shown in Figure 7h, MDA contents of all pork samples continuously increased with prolonged storage time. The MDA content of the control group exceeded 0.6 mg/kg after 4 days of storage, indicating slight meat lipid oxidation. After 10 days, the MDA value was higher than 1.0 mg/kg, demonstrating severe lipid oxidation of pork. Compared to the control group, PLA/PBAT-based composite films significantly suppressed lipid peroxidation in pork. Meanwhile, the MDA content of the PLA/PBAT/ZIF-8 group exceeded 0.6 mg/kg after day 6, demonstrating its certain antioxidant capacity in retarding lipid oxidation. As the ANTs@ZIF-8 content increased, the MDA content of the PLA/PBAT/ANTs@ZIF-8 group was significantly lower than that of the PLA/PBAT/ZIF-8 group at all storage time points, which indicated that the addition of ANTs effectively enhanced the antioxidant performance of the film matrix. Among all groups, the PLA/PBAT/3%ANTs@ZIF-8 group maintained MDA levels at approximately 0.6 mg/kg throughout the 12-day storage period. This indicated that ANTs could be effectively released over prolonged storage, inhibiting the formation of lipid oxidation products and lowering MDA accumulation. This phenomenon was attributed to the composite film’s ability to prevent oxygen contact with pork, thereby slowing down fat oxidation. This finding demonstrated that ANTs, as an excellent antioxidant-active molecule, combined with ZIF-8 nanoparticles, effectively retarded lipid oxidation of pork, thereby alleviating oxidative deterioration and improving meat preservation performance [53].

## 4. Conclusions

In this study, ANT with excellent antioxidant and antibacterial activities was encapsulated by ZIF-8, and a novel PLA/PBAT/ANTs@ZIF-8 composite preservation film was successfully prepared via solution casting method. The prepared film materials were systematically characterized by SEM, FTIR, XRD and TGA. The results showed that ANTs@ZIF-8 nanoparticles could be uniformly dispersed in the PLA/PBAT blend system, forming a dense film structure and achieving good composite effect with the matrix. The prepared PLA/PBAT/ANTs@ZIF-8 composite film showed good TS, EAB, and water vapor barrier properties as well as an excellent antibacterial effect against *E. coli* and *S. aureus*, which could meet the practical needs of pork packaging. Pork preservation tests showed that during the 12-day refrigerated storage period, the PLA/PBAT/3%ANTs@ZIF-8 composite film exhibited the optimal preservation effect, which could stably maintain the MDA content of pork at about 0.6 mg/kg, keep the TVB-N content below 15 mg/100 g, effectively maintain the color quality of pork, reduce juice loss, and significantly delay pork spoilage. In conclusion, the PLA/PBAT/ANTs@ZIF-8 composite film achieved the synergistic optimization of structure, mechanical properties and preservation performance. This green and environmentally friendly packaging material will have broad application prospects in the field of fresh food preservation.

## Data Availability

The original contributions presented in the study are included in the article. Further inquiries can be directed to the corresponding author.

## Supplementary Materials

The following supporting information can be downloaded at: https://www.mdpi.com/article/10.3390/molecules31172970/s1, Figure S1: SEM image of as-synthesized ZIF-8 nanoparticles.
