# Supplementary material for: Preparation of PLA/PBAT/Anthocyanins@ZIF-8 Composite Films via Casting Method and Their Antibacterial Activity and Application for Pork Preservation
Source: Molecules. 2026 Aug 25;31(17):2970. doi: 10.3390/molecules31172970 (PMC13567167; doi:10.3390/molecules31172970)
Supplement: Supplementary file 1 [file molecules-31-02970-s001.zip › molecules-4485040-supplementary.pdf]

Supplementary Information

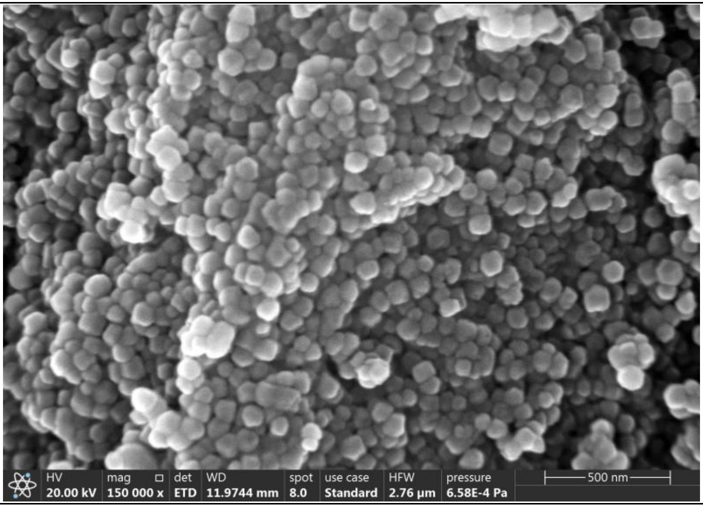

Figure S1. SEM image of as-synthesized ZIF-8 nanoparticles.
